# Changes in Migraine Headaches Following SARS-CoV-2 Infection: A Case Series

**DOI:** 10.7759/cureus.82708

**Published:** 2025-04-21

**Authors:** Neil Shah, Deep Patel, Amber Sousa, Adena N Leder

**Affiliations:** 1 Osteopathic Medicine, New York Institute of Technology College of Osteopathic Medicine, Old Westbury, USA; 2 Osteopathic Manipulative Medicine, New York Institute of Technology College of Osteopathic Medicine, Old Westbury, USA

**Keywords:** covid-19, migraine, neurology, pain, treatment

## Abstract

During the COVID-19 pandemic, changes in migraine headaches have been observed. This case series of seven patients seeks to identify changes in migraines following COVID-19 infection and discuss the mechanisms by which these changes may have occurred. The study was composed of seven subjects with prior COVID-19 infection and a preceding history of migraine disorder. Most of the subjects presented with increased frequency and intensity of migraines shortly or immediately following COVID-19 infection. Many also described a shift in pain from local to diffuse headache. Additionally, some subjects developed other neuropsychiatric symptoms consistent with long COVID that included brain fog and, unusually, aphasia. After COVID-19 infection, six subjects had reduced efficacy for their medications and had to alter their regimen. Standard treatments such as Botox and anti-calcitonin gene-related peptide (anti-GCRP) had varied success among the cases. It is important to note that the pathophysiology of migraines during COVID-19 is still unclear and other factors can play a role. Nevertheless, individuals with a history of migraines noticed worsening symptoms and changes in medication efficacy following COVID-19 infection.

## Introduction

Background of COVID-19 

Research has found that the ACE2 receptors expressed in the lungs, kidneys, heart, and several other organs serve as the cellular entry point for SARS-CoV-2 as the spike proteins show a high binding affinity to these receptors [1]. The virus can present itself asymptomatically or range from common cold-like symptoms to complete respiratory failure [1]. While some symptoms are temporary, others may persist long after infection, often termed "long COVID". These include various forms of headaches, including migraines, among a wide variety of other symptoms. 

Pathophysiology of migraines 

A migraine is a complex neurological dysfunction and can impact a person's quality of life. Migraines are characterized by bouts of unilateral headaches that can last a few hours to days and are often accompanied by nausea and heightened sensitivity to light and sound. Many triggers, such as stress, specific foods, environmental changes, and hormonal changes, can induce migraines. Due to the complexity of this condition, the underlying mechanism remains unclear. The proposed pathophysiology follows a cortical spreading depression of neuronal depolarization thought to activate trigeminal nerve afferents that lead to pain and the development of possible auras involved in migraines. 

Cortical spreading depression has been shown to release molecules such as nitric oxide, adenosine triphosphate (ATP), calcitonin gene-related peptide (CGRP), and glutamate. These substances are thought to diffuse towards the cortex of the brain and activate pial and dural nociceptors, triggering persistent neurogenic inflammation and pain. The sensitization of meningeal nociceptors is what is believed to cause the throbbing nature of migraine pain. Cephalic cutaneous allodynia during migraine is thought to be also mediated by the sensitization of central trigeminovascular neurons in the trigeminal nucleus caudalis, whereas extracephalic (whole-body) cutaneous allodynia during migraine is thought to be mediated by the sensitization of the central trigeminovascular neurons in the thalamus. Overall, migraines are thought to be mediated by the dysfunction of central nervous system (CNS) structures involved in the control of neuronal excitability and pain, as well as pain signals originating in peripheral intracranial nociceptors that activate the trigeminovascular pathway [2]. 

Migraines and COVID-19 

Headaches have been commonly associated with both acute COVID-19 infection and as a symptom of long COVID. These headaches are most often migraine-type or tension-type and have been observed to be worsened in individuals with pre-existing headaches [3]. In COVID-19 patients with headaches, higher levels of ACE2 receptors were observed, which is directly related to the entry point of the virus. Further, inflammatory markers HMGB1, NLRP3, and IL-6 were elevated in these patients. These molecules play a role in stimulating the trigeminovascular system hypothesized to cause headache pain [4]. 

In several studies, changes in migraines were associated with a COVID-19 infection. According to one study, an increase in antimigraine medications, an increase in the frequency of migraine attacks, and an increase in anxiety were observed [5]. Similarly, another study found that individuals with a prior history of migraines experienced longer and more intense headaches after COVID-19 infection [6]. In a self-administrable questionnaire in which 102 individuals with a confirmed COVID-19 diagnosis participated, more than half reported worsening in their migraines after COVID-19 infection, with increased frequency and significantly worse pain [7]. 

In this case series, we seek to identify persistent changes in migraines following COVID-19, patterns across participant experiences, other co-occurring neurological and psychological changes, and migraine treatments that may be more efficacious post-COVID-19. 

## Materials and methods

Participants

This case series includes seven participants recruited from the clinic of Dr. Adena N. Leder, a neurologist at the New York Institute of Technology College of Osteopathic Medicine in Old Westbury, New York. All participants signed an informed consent form for the utilization of their personal data and medical records. All data was recorded in an encrypted RedCap database. Each of the seven cases will be described in a de-identified format without any quantitative analyses. Approval to conduct the study was obtained from the New York Institute of Technology Biomedical and Health Sciences Research (BHS) Institutional Review Board (IRB) (approval number: BHS-2024-131).

Inclusion criteria for the study required participants to be 18 years or older, have been diagnosed with migraines prior to COVID-19 infection(s), and have been taking medications for their migraines before COVID-19 infection(s). They must have also noticed migraine changes post-COVID-19 (within six months of infection onset) and have had a reported positive COVID-19 test. The participants were confirmed to have COVID-19 via various diagnostic methods, including polymerase chain reaction (PCR) and rapid antigen test, reported by the participant and/or noted in their medical records. Participants were not eligible for this study if they had other known causes of migraine changes or if they were under 18 years old. All participants were interviewed with a standardized questionnaire that involved collecting basic demographic information, a thorough general medical history, and a history specific to their migraines and treatment. Pain level was evaluated using the visual analog scale. 

Tables 1-2 show the diagnostic criteria for migraines without aura and with aura, respectively.

**Table 1:** Diagnostic criteria for migraines without aura (ICHD-3) ICHD: International Classification of Headache Disorders Adapted from: [8]

Lasts 4-72 hours
At least two of the following:
Unilateral
Moderate to severe pain
Throbbing/pulsing
Aggravated by physical activity
At least one of the following:
Nausea or vomiting or both
Photophobia and phonophobia

**Table 2:** Diagnostic criteria for migraines with aura (ICHD-3) ICHD: International Classification of Headache Disorders Adapted from: [8]

Lasts 4-72 hours
One or more of these fully reversible aura symptoms:
Visual
Sensory
Speech and language
Motor
Brainstem
Retinal
At least three of the following six characteristics:
At least one aura symptom spreads gradually over five minutes or more
Two or more symptoms occur in succession
Each aura symptom lasts 5-60 minutes
At least one aura symptom is unilateral
At least one aura symptom is positive
The aura is accompanied, or followed within 60 minutes, by the headache

## Results

Case 1

Case 1 was a 34-year-old white woman with an early-onset history of migraines with aura from the age of 10 and anxiety diagnosed in 2021. For most of her life, she experienced primarily a gustatory aura in which the food she ate tasted unusual. Later, she began experiencing an olfactory aura where she describes the smell of flowers or trees blooming accompanying and/or preceding her migraines. Screen sensitivity was common with these migraines. Before COVID-19 infection, she experienced about 10 migraines a month at a severity rated 4/10, with very severe ones every two months. These attacks would last anywhere from one to three days and were described as throbbing in nature in the forehead region. She was prescribed Aimovig 70 mg and took it once a month and amitriptyline once daily. The migraines did not have any reported significant impact on her functioning, but they were painful, and she felt irritable. After a COVID-19 infection in May of 2022, the frequency of her migraines increased from about 10 to approximately 14, with the more severe migraines occurring every 4-6 months. Several of these would last upwards of three days and were rated a 5/10 or more in intensity. Additionally, she described the pain as more headband-type pulsing pain. The gustatory and olfactory auras continued. Additionally, the participant admitted to increased screen sensitivity and brain fog with some aphasia occurring with severe migraine attacks. She was started on Botox injections (200 units) every three months after her COVID-19 infection, in addition to continuing her previous medications. She stated that the addition of Botox made her migraines manageable again. Currently, she deals with 15 migraines per month lasting a few hours up to three days, with at least 10 being low severity, attributed to her new Botox treatment. 

Case 2

Case 2 was a 74-year-old white man with worsening migraine symptoms since his COVID-19 infection in June 2024. He reported a long history of migraines that started in his late teens and other medical comorbidities such as heart disease, irritable bowel syndrome (IBS), acid reflux, and atrial fibrillation. Before his COVID-19 infection, his migraines occurred 14 times per month, lasting from two to six hours, with a severity of 5-6/10. He felt pain around his left eye that radiated throughout his head. The patient complained of nausea but did not experience light or sound sensitivity. He was treated with metoprolol 200 mg, rosuvastatin 20 mg, Xarelto 20 mg, CoQ10 300 mg, vitamin D3 5000 IU, and silodosin 8 mg daily. In addition, he received Botox injections every three months and Aimovig 140 mg once a month for migraine treatment. For severe migraine attacks, he used Zomig spray 5 mg and Relpax 40 mg as needed. His migraines moderately impacted his daily activities. After a COVID-19 infection, the patient's symptoms were exacerbated. He reported migraines 26 times per month, lasting over six hours, with a severity of 6-7/10. He describes the pain as diffuse rather than localized to the left eye, still radiating throughout the head. Due to the increase in migraine attacks, the patient replaced Aimovig 140 mg monthly with Quilipta 60 mg monthly while still adhering to the other medications. He reports an increasing dependence on Zomig spray 5 mg and Relpax 40 mg. The migraines are not well-controlled despite the adjustments to the medication regimen, and there is reduced efficacy of the treatment plan. The patient often feels depressed and discouraged as a result of the migraines affecting the quality of his physical and mental well-being. Currently, he experiences approximately 17 migraines per month lasting 2-6 hours on average. 

Case 3

Case 3 was a 61-year-old Hispanic woman with migraines of increasing frequency and intensity since contracting COVID-19 in December 2021. She has a history of chronic migraines since age 20 in which she would experience around 10 migraine episodes a month. She reported a history of breast cancer and has had a mastectomy and three lymph nodes removed in May 2021. Just before 2021, due to her great response to Botox treatment, she experienced 0-1 migraine episode per month that lasted 7-24 hours, with localized pain rated at 3/10. Her migraines were associated with nausea, vomiting, dizziness, and sensitivity to light, sound, and perfume, with no aura. The patient's treatment consisted of Botox injections every three months, which significantly improved the frequency and severity of her attacks. She also used sumatriptan 50 mg and sumatriptan/naproxen (Treximet) 85-500 mg as needed. She stated Botox was highly effective in reducing her migraines and decreasing her pain to a 3/10. However, after the COVID-19 infection, the frequency of the patient's migraines increased from once a month to twice a month, lasting 24-48 hours, with severe pain reaching 7-8/10 at its worst. She described the pain as diffuse rather than localized. Her prior medication regimen, including Botox, was no longer effective. She relies on sumatriptan 50 mg and Treximet 85-500 mg more often, yet experiences minimal relief. She reported feeling unmotivated and disheartened, and she has been gaining weight due to the mental stress of her migraines. She is also suffering from an increase in body aches, falls, neurological symptoms resembling attention-deficit/hyperactivity disorder (ADHD), a "stuffy head", and stomach discomfort upon waking up, which were not present before the infection and have not been attributed to any other causes. Currently, she deals with migraines twice a month lasting three to four days despite her ongoing Botox treatment. 

Case 4

Case 4 was a 32-year-old white man with a history of migraines since age 14 and worsening migraines since a COVID-19 infection in January 2021. Before the infection, the patient reported one migraine per month, lasting 6-8 hours, rated at 6/10 for discomfort and pain. He described the pain as severe and pulsating, extending from the temporalis and occipitalis to the frontalis. He experienced photophobia, nausea, vomiting, and light sensitivity. The patient historically used triptans and increased his water intake during attacks but felt little relief from these interventions following his infection. He described himself as physically active and healthy, adhering to a Mediterranean and gluten-free diet. The migraines were debilitating and prevented him from engaging in hockey, weightlifting, and kickboxing. Post-COVID-19, the patient reported no change in the number of migraines per month, still enduring one per month. However, the attacks have a longer duration, 6-12 hours, with a pain rating of 8/10. He takes Excedrin 325 mg as needed in addition to the triptans but sustains worsening photophobia, nausea, vomiting, and light sensitivity. He feels as though his migraines have been a significant burden on his lifestyle as a result. 

Case 5

Case 5 was a 74-year-old white woman with a history of migraines with no auras since the age of 28. Her comorbidities included hypertension and heart disease. She experienced extreme light sensitivity with these migraines, and her triggers included temperature and humidity changes. She reported nausea but no vomiting. Prior to her COVID-19 infection, she would experience migraines 4-6 times a month lasting several hours, rated at 5/10 in severity. She described the pain as throbbing in her temples and the top of her skull, as if being pierced by a jackhammer. She denied any auras or other sensations prior to her migraine attacks. She took Excedrin and Tylenol as needed with over-the-counter (OTC) doses for the migraines and was able to function well despite the attacks, with no significant impact on her daily activities. After contracting COVID-19 in June 2022, she noticed an increase in migraine attacks upwards of six per month with the severity and pain also increasing to an 8 or 9/10. Since then, she has described an almost constant pain in her head that gets better and worse at different times. She reported that the pain is more concentrated in her forehead over her eyes where the frontal sinuses are located. The pain is described as a tightening sensation, and she admits to increased light sensitivity, especially when watching television. She stated that she is unable to function as well as before, complaining of feeling like she is much older, affecting her ability to do daily house chores and drive. Despite OTC medications, she still suffers from six or more migraines a month with almost constant head pain. 

Case 6

Case 6 was a 35-year-old white woman with migraine onset at 28 years old. She was also diagnosed with Parkinsonian-like syndrome, with dystonia and dyskinesia in 2017. She experienced nausea and vomiting, as well as light sensitivity with her migraines, but no auras. Pre-COVID-19 infection, her migraines would occur once a month, usually in line with her menstrual cycle, and they would last a few hours. She described these as moderate in pain rating them as a 6/10. The pain was throbbing and localized primarily to her forehead. She took Tylenol for pain relief but reported that the migraines would affect her sleep negatively and would regularly cause her to feel nauseous and vomit. After contracting COVID-19 in July 2022, her migraines increased to 2-3 times a month lasting for several more hours than before. These also increased in severity, rating them an 8/10 on a subjective pain scale. She was prescribed EC-naproxen delayed-release 500 mg and Ubrelvy 100 mg for her migraines as needed. However, she admitted that she would only take Ubrelvy for intolerable headaches from migraines, while she would take naproxen for mild to moderate migraines. These migraines have had a greater effect on her sleep, making it difficult to fall asleep and causing sleep disturbances. Further, it has reduced her ability to exercise. Currently, she continues to have migraines 2-3 times a month lasting several hours despite being prescribed the new medications. 

Case 7

Case 7 was a 61-year-old white woman with a history of migraines since her early 20s. She denied any comorbidities. Her migraines would frequently occur with a visual aura that included spots on the periphery of her vision. Further, she experiences nausea and light sensitivity. Before her COVID-19 infection, she reported having one migraine every few months, lasting up to a full day. She rated the pain a 10/10 in severity, with the pain being frontal and ocular in region, concentrated around her forehead. She was prescribed Imitrex and Ubrelvy, neither of which worked to relieve the migraines, and used Advil 200 mg instead as needed for the pain. She stated that the migraines were debilitating and made it difficult to do basic activities like work and house chores. After a COVID-19 infection in December 2022, her migraines became more frequent with one or more per month. She also believed that the COVID-19 infection was a direct trigger for her migraines, having contracted the virus multiple times and having a migraine occur each time concurrently. These migraines would be 2-3 days long and would be just as severe, if not more, as before COVID-19, rating them a 10/10. The pain is still frontal and ocular, and she admits to the same aura and sensitivities as before. She is prescribed Botox for her migraines at this time, once every three months, which seems to be the only form of treatment that has been effective. As a result, her migraines have become much less frequent, with only one migraine every few treatments of Botox, and not severe at all. 

Table 3 provides a summary of the clinical data recorded from the participants.

**Table 3:** Summary of the clinical data recorded from the participants

Case	Age	Sex (M/F)	Race	Infection date	Migraine changes	Effective treatments (post-COVID-19)	Ineffective treatments (post-COVID-19)
1	34	F	White	May 2022	Increased frequency, severity, and duration	Botox	Amitriptyline
					Increased screen sensitivity		
					Brain fog and aphasia with severe attacks		
							Aimovig
					Change from throbbing to headband-pulsing		
2	74	M	White	June 2024	Increased frequency, severity, and duration	Zomig spray	Metoprolol
					More diffuse pain rather than localized around the eye		Botox
							Quilipta
						Relpax	
					Depressive symptoms		
3	61	F	Reported more than one race	December 2021	Increased frequency, severity, and duration	N/A=no relief	Botox
					More diffuse pain rather than localized		
							Sumatriptan
							Sumatriptan/naproxen (Treximet)
					Increase in body aches, falls, ADHD-like symptoms, stomach upset		
4	32	M	White	January 2021	Increased severity and duration	N/A=no relief	Triptan
					Worsened associated symptoms (photophobia, N/V)		
							Excedrin
5	74	F	White	June 2022	Increased frequency and severity	N/A=no relief	Excedrin
					Almost constant head pain		
					Pain is tightening rather than throbbing and concentrated around the frontal sinuses		
							Tylenol
					Increased light sensitivity		
6	35	F	White	July 2022	Increased frequency, severity, and duration	N/A=no relief	Tylenol
							EC-naproxen delayed-release
							Ubrelvy
					Reduced sleep and ability to exercise		
7	61	F	White	December 2022	Increased frequency, severity, and duration	Botox	Imitrex
							Ubrelvy
							Advil

## Discussion

Summary of clinical patterns and distinctions 

Across the cohort of cases presented, all participants except for Case 4 experienced an increase in both the frequency and severity of their migraines and reduced efficacy of prior treatments. The worsening of migraines after COVID-19 infection is hypothesized to be due to a direct viral effect on the trigeminal vascular system [9]. COVID-19 is known to cause a cytokine storm that may lead to the cytokine-mediated activation of trigeminal nerve endings and/or direct invasion of nerve endings by SARS-CoV-2 [9]. Another model is proposed to involve endothelial cells of blood vessels that highly express ACE2 in the activation of the trigeminal vascular system [9]. 

A key observation from these case studies is a clear evolution of migraine characteristics. These include neurological manifestations and changes in pain localization and associated symptoms. Participant 1 notably began to have brain fog and some aphasia with severe migraine attacks following her COVID-19 infection. Although neuropsychiatric symptoms have been associated with COVID-19 infection, these have commonly included headaches, agnosia, and anosmia, among others. Aphasia is rarely written about in literature in association with COVID-19 and migraines. One case study specifically mentions headache with neurological deficits and cerebrospinal fluid (CSF) lymphocytosis (HaNDL) syndrome seen in a 14-year-old male patient after COVID-19 infection [10]. He presented with three episodes of a migraine-like headache including nausea, photo-/phonophobia, and language deficits. Diagnosis was confirmed with CSF analysis revealing lymphocytic pleocytosis with otherwise clear results. The syndrome is often preceded by a viral illness, in this case, with SARS-CoV-2. Brain fog is a common symptom of long COVID and has been associated with elevations of CCL11, a specific cytokine that was found to be increased in the plasma of COVID-19 patients who presented with cognitive dysfunction [11]. 

Additionally, participant 3 admits to experiencing an ADHD-like disorder after a COVID-19 infection. A study analyzing the most common long-term effects of COVID-19 found that attention disorder was among the top five reported symptoms post-infection [12]. Attention disorders like ADHD have long been linked to imbalances in neurotransmitters, primarily a reduction in dopamine [13]. This aligns with a 2024 study that discovered dopamine neurons are susceptible to infection by SARS-CoV-2, which triggers early senescence in these neurons, leading to cell death and inactivity and presumably a decrease in dopamine release [14]. Several participants also described a shift in pain location with Cases 1 through 3 experiencing a change from localized pain ranging from forehead to periorbital to more general, diffuse pain in their head during their migraines. However, those participants who also had an aura before their migraines did not experience any changes in their aura(s). 

Finally, the psychological and functional impact of worsening migraines in many of our participants is profound and significant. Participant 2 describes feelings of depression and being discouraged, with participant 3 reporting similar symptoms of emotional distress including feeling demotivated. Participants 4 and 7 report that the worsened migraines have diminished their ability to do basic activities like house chores. Participant 4, specifically, admits she feels the effects of age much more now. Participant 5 describes the effects of his worsening migraines post-COVID-19 as a significant impediment to his lifestyle, while participant 6 mentions negative effects on her sleep, including insomnia, and particularly on her ability to exercise which she uses to manage her Parkinsonian-like syndrome. Given the frequency of such psychological impacts, assessment and referral for psychological treatments should be part of migraine management post-COVID. 

Migraine treatment 

Historically, treatment for migraine attacks has consisted of lifestyle changes, exercise, and medications. There are many medication options available, but none are ideal for all patients. Abortive treatment options for migraine include nonsteroidal anti-inflammatory drugs (NSAIDs), acetaminophen, triptans, CGRP antagonists, antiemetics, and dihydroergotamine. These extensive classes of medications are often used together and are evidence-based options for acute migraines [15]. In 2018, a review article was published providing current prophylactic treatments for migraine and their various mechanisms of action. These included beta-blockers, antiepileptics, calcium antagonists, antidepressants, and onabotulinumtoxin-A. The potential mechanisms for these classes of drugs include suppression of the cortical spreading depression, reduction of excitatory glutamatergic neurotransmission, and facilitation of inhibitory GABAergic neurotransmission [16]. 

A common similarity found in all seven cases was that migraines became more severe and less manageable by their prior medication treatment after a COVID-19 infection. Additional medications were added to the regimen for six of the seven cases. Case 7 found her initial treatment regimen of Imitrex (sumatriptan), Ubrelvy (ubrogepant), and Advil to be less effective before her COVID-19 infection. After her COVID-19 infection, she was switched to only Botox (onabotulinumtoxin-A) every three months and noticed her migraines became more tolerable. In parallel, Case 1 also found that her migraines following COVID-19 became more manageable once Botox was added to her initial regimen of amitriptyline and Aimovig (erenumab). Case 7 had similar success with Botox. In contrast, Case 3 had an already effective migraine regimen that included Botox. However, after infection, Botox was noted as not as effective, and medications like NSAIDs and sumatriptan were largely used thereafter. 

Because the underlying mechanisms for migraine changes following SARS-CoV-2 infection have not been elucidated, it is not yet possible to direct treatment from this knowledge. In our small study, it does appear that Botox treatment offers a potential treatment addition for some patients. Currently, medications are being researched on their effectiveness for migraines after COVID-19 infection. A recent 2024 study on an Italian subpopulation found CGRP antagonists to have greater efficacy [7]. Some data suggests that there may be a connection between COVID-19 and higher serum levels of CGRP [7]. Cases 1, 2, and 6 have implemented anti-CGRP as their current migraine regimen post-COVID-19. Although efficacy has not been recorded yet by these cases, a follow-up and further research into the efficacy of this medication is warranted. 

Additionally, associated symptoms of migraines were found to be less effectively managed by medications after COVID-19. For instance, Case 4 was started on Excedrin for migraine attacks but still noted worsening photophobia, nausea, vomiting, and light sensitivity. Similarly, Case 5 found that her migraines became less tolerable with an additional increased light sensitivity. 

Finally, many of the cases experienced debilitating effects in their daily activities and lifestyle. Many noted mental health changes, while others found that they became unable to function as effectively. Due to the recent COVID-19 pandemic, lifestyle modifications have become a focus of intervention alongside medications when treating post-COVID-19 migraines. Low levels of physical activity and obesity are associated with an increased frequency of migraines. Furthermore, having an aerobic exercise program has shown a similar effect on migraines to the antiepileptic medication, topiramate, in reducing migraine pain intensity and frequency [17]. Several cases have incorporated exercise in their routine, and some have adjusted their diets. While there is no specific diet for migraine management, a weight-loss diet is recommended when appropriate. Additional factors that could reduce migraine attacks include limiting alcohol, caffeine, and nicotine [17]. Overall, lifestyle changes along with avoidance of triggers are important to managing migraines, and this remains true post-COVID-19 where stress levels and psychiatric comorbidities have been found to rise. 

Future directions 

It is important to recognize patients who develop new migraines after COVID-19 infection, as our cases had an already known migraine diagnosis before COVID-19 infection. Further longitudinal study is needed to investigate the long-term effects of COVID-19 on migraine pattern, severity, frequency, and response to therapy. Further studies are recommended to identify the mechanism of the effect of COVID-19 infection on migraine pathophysiology including cortical spreading depressions and the trigeminovascular pathways. Also, future research should look at the presentation of migraines at all ages including children and adolescents. It is also important to note the various extrinsic factors that may be in part leading to the presentations seen in these cases. 

Factors like stress and diet could play an important role in migraine episodes especially during the COVID-19 pandemic. An anonymized self-reported data study was able to look at these components in the United States using a migraine diary smartphone application, Migraine Buddy. In this study, participants reported COVID-19-related stress as the most common source of stress that was attributed to their migraines during the pandemic. In addition, participants reported experiencing significant life changes in a short amount of time which resulted in difficulty in managing migraine-friendly diets, scheduling doctor appointments, and accessing health and emergency services [18]. Thus, it is essential to recognize that migraine presentations, including the ones seen in this case series, could be attributed to other factors such as diet, lifestyle, and stress. More research is needed to examine both the direct effects of SARS-CoV-2 on migraine and the indirect effects of COVID-19 on migraine and its management. Treatments for post-COVID-19 migraine need further study.

## Conclusions

The significant neurological and emotional burden the participants experienced post-COVID-19 due to the worsening of their migraines often impacted their quality of life. Participants reported changes in pain localization, increased migraine frequency and severity, and additional neurological manifestations that included brain fog and aphasia. The emotional toll of these worsening symptoms was also evident for some, with participants reporting depression and a lack of motivation with a reduced ability to perform daily activities. Although these symptoms, coupled with reduced treatment efficacy, underscore the far-reaching impact on these participants' lives, this study is constrained by some limitations. These include the small sample size and the lack of imaging data to investigate any neuroanatomical changes. Because data was collected via medical record review and self-report history, there are issues of recall bias and nonuniformity of data collection methods. Nevertheless, this case series highlights the complex changes seen in migraine patterns post-COVID-19, illustrating the need for more thorough research and implementation of additional treatment solutions. 
